# Differential Effects of Green Tea Powders on the Protection of Brown Tsaiya and Kaiya Ducklings against Trichothecene T-2 Toxin Toxicity

**DOI:** 10.3390/ani11092541

**Published:** 2021-08-30

**Authors:** Ko-Hua Tso, Chompunut Lumsangkul, Min-Chien Cheng, Jyh-Cherng Ju, Yang-Kwang Fan, Hsin-I Chiang

**Affiliations:** 1 Department of Animal Science, National Chung Hsing University, Taichung 40227, Taiwan; d100037004@mail.nchu.edu.tw (K.-H.T.); mccheng@mail.tlri.gov.tw (M.-C.C.); 2 Department of Animal and Aquatic Sciences, Faculty of Agriculture, Chiang Mai University, Chiang Mai 50200, Thailand; chompunut.lum@cmu.ac.th; 3 Hengchun Branch Institute, Livestock Research Institute, Council of Agriculture, Pingtung 94644, Taiwan; 4 Graduate Institute of Biomedical Sciences, China Medical University, Taichung 40402, Taiwan; 5 Translational Medicine Research Center, China Medical University Hospital, Taichung 40402, Taiwan; 6 Department of Bioinformatics and Medical Engineering, Asia University, Taichung 41354, Taiwan; 7 Center for the Integrative and Evolutionary Galliformes Genomics, National Chung Hsing University, Taichung 40227, Taiwan

**Keywords:** Brown Tsaiya, green tea powder, growth performance, Kaiya duck, plasma biochemical parameters, T-2 toxin

## Abstract

**Simple Summary:**

The objective of this study is to examine the effects of T-2 toxin (T-2) and green tea powders (GTP) on growth performance, hematology, and pathology parameters in Brown Tsaiya ducklings (BTDs) and Kaiya ducklings (KDs). T-2 toxin shows a strong and differential toxicity in growth suppression, as well as abnormalities in the hematological and pathological parameters of BTDs and KDs. We found that GTP could potentially prevent T-2-induced poor growth performance and improve some hematological parameters. Moreover, BTDs were more sensitive than KDs in terms of responses to T-2 toxicity and GTP detoxification.

**Abstract:**

A 3-week feeding trial in a 3 × 2 × 2 factorial design was conducted with three concentrations (0, 0.5, and 5 mg/kg) of T-2 toxin (T-2) and two levels (0% and 0.5%) of green tea powder (GTP) supplements used in the diets of female brown Tsaiya ducklings (BTDs) and Kaiya ducklings (KDs), respectively. Breed had a significant effect on the growth performances and the relative weights of organs and carcass. In general, the growth performances of KDs were better than BTDs. The relative weights of organs and carcass of BTDs were typically heavier than those of KDs; however, the breast of KDs was heavier than those of BTDs. Both ducklings received 5 mg/kg of T-2 blended in the diet showed lower feed intake and body weight gain (BWG) in the second and the third week. The diet containing 5 mg/kg of T-2 and 0.5% GTP improved the BWG compared to those fed the diet supplemented with 5 mg/kg of T-2 without GTP in BTDs. Ducklings fed the diet containing 5 mg/kg of T-2 induced hypocalcemia and hypomagnesemia, as well as decreased concentrations of creatine phosphokinase and alkaline phosphatase. The concentrations of blood urea nitrogen (BUN) and glutamate oxaloacetate transaminase (GOT) were increased in KDs and BTDs fed the diet containing 5 mg/kg of T-2 without GTP, respectively. However, duckling diets containing 5 mg/kg of T-2 with 0.5% GTP lowered concentrations of BUN and GOT in the blood plasma of KDs and BTDs, respectively. The diet containing 5 mg/kg of T-2 increased the relative kidney weight but decreased the relative breast weight of ducklings. Enlarged gizzards and reduced relative leg weights were observed in BTDs fed the diets containing 5 mg/kg of T-2. In summary, BTDs are more sensitive than KDs in responding to T-2 toxicity and GTP detoxification. Green tea powder has detoxification ability and could potentially mitigate T-2 toxicity on BWG, BUN, and GOT in ducklings.

## 1. Introduction

Mycotoxins are secondary toxic metabolites produced by various mold species [1,2]. One of the most studied mycotoxins is trichothecenes, which has more than 200 derivatives [3]. The major chemical features responsible for the biological activities of trichothecenes are the 12,13-epoxy ring and the variable structures of side-chain branches, which determines the toxicities and characteristics among the different trichothecenes [4,5,6]. Trichothecenes are classified into four types (A, B, C, and D) according to their side-chain structures [6]. Within them, Type A includes T-2 toxin (T-2) and HT-2 toxin (HT-2), and type B includes deoxynivalenol (DON) and nivalenol (NIV). These two types of trichothecenes contribute the major problems of mycotoxin contamination in ingredients and feeds, mainly by T-2 and DON. T-2 toxin is a *Fusarium*-derived trichothecene, which can inhibit protein synthesis [7], induce lipid peroxidation [8], and damage the organs and gastrointestinal system in animals [9]. T-2 toxin can induce poor growth performance, but considerable differences exist between chickens and ducks in dosage, age, and response time. Kutasi et al. [10] reported that 1-day-old white Pekin ducklings decreased body weight by consuming feed contaminated only with 0.6 mg/kg of T-2 for 4 weeks, whereas in 1-day-old chicks, it took 3 weeks to show the reduced body weight after consuming feed contaminated with 4 mg/kg of T-2 [11]. It is known that waterfowls have more unsaturated fatty acids (UFA) in their body tissues than chickens [12]. Given that mycotoxin T-2 is fat-soluble, it can accumulate in animals’ bodies for months and make waterfowls more susceptible to the UFA-related damage than chickens [13]. Fernye et al. [14] also indicated that waterfowls were exceptionally sensitive to T-2. It has been reported that energy metabolism can be inhibited by T-2 in poultry [15]. Additionally, the basal metabolic rate differs significantly between meat-type and laying-type animals during their brooding period [16]. It is not known whether meat-type ducklings and egg-type ducklings are similarly sensitive to T-2 toxicity.

The chemical and physical properties of T-2 include high heat stability [4], high molecular weight, and low polarity [17]; therefore, the efficacy of detoxifying T-2 via thermal inactivation or absorbent binding is relatively limited [18]. T-2 toxin exhibits its toxicity mainly by inducing excessive production of free radicals, which trigger lipid peroxidation in animals [19]. It has been well-known that antioxidants, such as ascorbic acid, tocopherol, and selenium, can neutralize superoxide anion, reactive oxygen species (ROS) scavengers, free radicals, and lipid peroxidation by T-2 [20]. It is also reported that the antioxidants detoxified T-2 toxicity in poultry [21,22].

Recently, there has been increasing interest in finding natural antioxidants from plant phytochemicals to protect animals against free radicals [23,24]. Green tea is one of the most widely consumed beverages in Asian countries, such as China, Japan, India, and Taiwan. Green tea powder contain many functionally active substances, including polyphenols, catechins, alkaloids, and polysaccharides [25], which have attracted considerable attention as animal feed additives in recent years [26,27]. The antioxidant capacity of GTP comes from green tea polyphenol compositions [28], which include catechin (C), catechin gallate (CG), epicatechin (EC), epicatechin gallate (ECG), epigallocatechin (EGC), epigallocatechin gallate (EGCG), gallocatechin (GC), and gallocatechin gallate (GCG) [29]. The green tea polyphenol scavenges ROS and free radicals through several proposed mechanisms, including depolarization of electrons, formation of intramolecular hydrogen bonds, and rearrangement of molecular structure [30,31]. They interrupt oxidative reactions by chelating free copper and iron, which also catalyze the formation of ROS in animals [32]. Similarly, the antioxidant nature of green tea polyphenol is beneficial in preventing lipid metabolism disorders and reducing DNA damage [33,34,35]. Serving as an antioxidant, it has also been shown that GTP could detoxify the hepatotoxicity and genotoxicity of AFB_1_, fumonisin B_1_ (FB_1_), and citrinin [29,36,37] in different animal species. However, the effect of GTP on detoxifying T-2 remains elusive.

Brown Tsaiya ducks (BTDs) and Kaiya ducks (KDs) are endemic and popular laying- and meat-type duck species, respectively, in Taiwan. However, no reports on their response to T-2 toxicity are currently available. Green tea has long been recognized for its antioxidative potential in many aspects, which are thought to be beneficial in scavenging cellular ROS toxicity, a partial contributor associated with the T-2 toxicity. Therefore, the objectives of the present study are to test the effects of trichothecene T-2 along with GTP as a dietary supplement in duckling rations on the growth performance, plasma biochemical parameters, and certain representative organs weights of BTDs and KDs.

## 2. Materials and Methods

### 2.1. Animal Care and Use

This in vivo study was conducted in strict accordance with the guidelines recommended and approved by the Institutional Animal Care and Use Committee (IACUC) of National Chung Hsing University (Approval number: IACUC-100-41).

### 2.2. Production and Analysis of T-2 Toxin

T-2 toxin was produced by the fermentation of corn powder using *Fusarium trincintum*, which was selected from mold-contaminated wheat. Corn was inoculated with *F. trincintum* (approximately 10^8^ conidia/mL) conidia via dispersion and placed in Erlenmeyer flasks. After culturing at 14 °C in the dark for 28 days [38], the culture was dried at 65 °C for 2 days. The dried culture was crushed into powder using a shredder (RT-UF26, Rong Tsong, Taichung, Taiwan). Quantification of T-2 in inoculated corn powder was performed by high-performance liquid chromatography (HPLC) following the extraction, purification, and analysis based on the protocol established in Visconti et al. [39] with minor modification. Briefly, standards and sample extracts were injected into an HPLC pump (L2130, Hitachi, Tokyo, Japan) with a fluorescence detector (L-2485, Hitachi, Tokyo, Japan) using an auto-sampler (L-2200, Hitachi, Tokyo, Japan) set to an excitation wavelength of 381 nm and an emission wavelength of 470 nm. The flow rate of the mobile phase was set at 1.0 mL/min. A binary gradient was applied as follows: The initial composition of the mobile phase was established; 70% acetonitrile/30% water for 5 min, after which the acetonitrile content was increased to 85% in 10 min and kept constant for 7 min. Finally, the acetonitrile was decreased to 70% in 1 min and kept constant for 7 min. Acetonitrile was HPLC grade and purchased from Sigma (Saint Louis, MO, USA). The HPLC Mightysil^TM^ RP-C18 column (4.6 mm × 250 mm, 5 μm, Kanto Chemical, Tokyo, Japan) was used in the analysis. The concentration of T-2 in the corn powder was 230 mg/kg.

### 2.3. Compositions and Antioxidative Capacity of Green Tea Powder

#### 2.3.1. Analyses of the Chemical Composition and Catechin Concentration

Green tea powder was provided as a commercial feed additive by Leshan Yujia Tea Development Co., Ltd. (Sichuan, China). Dry matter was analyzed according to AOAC methods [40]. Crude fiber, neutral detergent fiber, and acid detergent fiber were determined by using a fiber analyzer (ANKOM 200/220, ANKOM Technology, Macedon, NY, USA) according to Fay et al. [41]. Green tea polyphenols (C, CG, EC, ECG, EGC, EGCG, GC, and GCG) were determined by HPLC, as modified by Zhang et al. [42]. Briefly, the GTP sample (3 g) were dissolved with 150 mL water in water bath at 80 °C for 5 min for the extraction of polyphenols. The final solution was aliquoted to 150 mL and filtered (pore size 0.45 μm) for HPLC analysis. For the HPLC analysis of catechin, standards and samples were injected into an HPLC pump using an autosampler with an ultraviolet (UV) detector (L-2400, Hitachi, Tokyo, Japan) set to 280 nm. The flow rate of the mobile phase was 1.0 mL/min. The mobile phase consisted of water with 1% (*v*/*v*) formic acid (Saint Louis, MO, USA) and acetonitrile with linear gradient elution. The acetonitrile level was increased from 4% to 18.7% in 42 min, and then the acetonitrile level was decreased from 18.7% to 4% in 1 min. The analytical HPLC column used was Mightysil^TM^ RP-C18 column. The chemical compositions and catechin concentrations of GTP are shown in Table 1.

#### 2.3.2. Antioxidative Capacity of Green Tea Powder

The scavenging activity of 1,1-diphenyl-2-picrylhydrazyl (DPPH) radical was determined according to the method described by Villano et al. [43]. Briefly, a 25 mg/L solution of DPPH (Alfa Aesar, Haverhill, MA, USA) radical solution in methanol (Merck, Darmstadt, Germany) was prepared, and 1.95 mL of this solution was mixed with 50 μL of extract solution (100 to 4000 μg GTP/mL distilled water). The solution was then mixed by a vortex mixer (Vortex-Genie 2, Scientific Industries, Bohemia, NY, USA) and left for 90 min at room temperature in the dark. The absorbance (A) was measured at 515 nm by using a spectrophotometer (Smart Spec Plus, Bio-Rad, Hercules, CA, USA). Ascorbic acid (vitamin C, Vit. C, Sigma-Aldrich, Saint Louis, MO, USA) was used as a positive control. This activity was given as the percent of DPPH scavenging and was calculated as follows:

DPPH scavenging (%) = [(A_control_ − A_sample_)/A_control_] × 100 (1)

where A_sample_ is the absorbance of sample containing GTP and A_control_ is the absorbance of the sample without GTP.

Total reducing power was determined according to the method described by Yildirim et al. [44]. Briefly, GTP (100 to 4000 μg) was dissolved in 1 mL of distilled water, then mixed with 1 mL of 0.2 M phosphate buffer (pH 6.6) and 1 mL of 1% potassium ferricyanide (Sigma-Aldrich, Saint Louis, MO, USA), after which the mixture was incubated at 50 °C for 30 min. Next, 1 mL of 10% trichloroacetic acid (Sigma-Aldrich, Saint Louis, MO, USA) was added, and the mixture was centrifuged at 3000× *g* for 10 min. Finally, 1 mL of upper layer solution was mixed with 1 mL of distilled water and 0.1 mL of 0.1% ferric chloride (Sigma-Aldrich, Saint Louis, MO, USA). The solution was then mixed by a vortex mixer and left for 10 min at room temperature in the dark. The absorbance was measured at 700 nm by a spectrophotometer. Vitamin C was used as a positive control. The absorbance values correspond to the capacity for metal ion reduction.

Ferric chelating activity was determined according to the method described by Dinis et al. [45]. Briefly, the extracts (100 to 4000 μg) in 1 mL of distilled water were mixed with 3.7 mL of methanol and 0.1 mL of 2 mM ferrous chloride (Sigma-Aldrich, Saint Louis, MO, USA) and then left for 30 s. Next, 0.2 mL of 5 mM ferrozine (Sigma-Aldrich, Saint Louis, MO, USA) was added. The solution was then mixed by a vortex mixer and left for 10 min at room temperature in the dark. The absorbance was measured at 562 nm by a spectrophotometer. Ethylenediaminetetraacetic acid (EDTA, Sigma-Aldrich, Saint Louis, MO, USA) was used as a positive control. The calculation of ferric chelating activity was shown as follows:

Ferric chelating activity (%) = [(A_control_ − A_sample_)/A_control_] × 100 (2)

where A_sample_ is the absorbance of sample containing GTP and A_control_ is the absorbance of sample without GTP.

### 2.4. Experimental Designs and Feeding Management of Ducklings

During the 3-week brooding period of BTD and KD, all ducklings were fed the same basal diet supplemented with various levels of T-2 and/or GTP as follows: basal diet without T-2 and GTP (control group), basal diet with GTP (0.5% GTP), low T-2 diet (0.5 mg T-2/kg), low T-2 diet with 0.5% GTP, high T-2 diet (5 mg T-2/kg), and high T-2 diet with 0.5% GTP. The T-2 concentration was designated to show the phenotypic T-2 toxicity and the experimental period was the length of the regular brooding period [10,11,46]. Moreover, 120 1-day-old female BTDs were obtained from the hatchery of the Department of Animal Science, National Chung Hsing University (Taichung, Taiwan). Another 120 1-day-old female KDs were obtained from Yilan Livestock Research Institute (Yilan, Taiwan). Ducklings of each type were randomly divided into six groups with four replicates per group (five ducklings in each replicate). The ducklings were fed ad libitum, with free access to clean water under light and heat supplied 24 h a day at an ambient temperature of 28 °C. To avoid synergistic effects caused by the contaminations of other mycotoxins, the basal diet was analyzed for AFB_1_, DON, FB_1_, HT-2, ochratoxin A (OTA), T-2, and zearalenone (ZEN) by HPLC. All of these mycotoxins had concentrations below the limit of detection (LOD < 10 μg/kg). The nutrients and chemical compositions of basal diet are shown in Table 2. The fungal powder of *F. trincintum* was mixed with the mycotoxin-free corn in the basal diets and adjusted to 0.5 or 5 mg/Kg of T-2 concentrations in the diets according to the experimental design. The GTP was additionally supplemented into the T-2 containing basal diets according to the experimental design. All duckling feeds were stored at 4 °C until use.

### 2.5. Growth Performances

During the experimentation period, body weights of day-old ducklings were recorded individually prior to the onset of the experiment and at the end of each week to calculate daily body weight gain (BWG, g/bird/day). Feed intake was recorded daily. The formulation for feed intake was as follows:

Feed intake = (Feed supplied − Feed remained)/Number of ducklings per replicate(3)

Feed conversion ratio (FCR) was calculated as feed intake (g/bird/day)/BWG.

### 2.6. Relative Weights of Organs and Carcass, and Plasma Biochemical Parameters

When the ducklings were 3 weeks old, 12 ducklings were randomly chosen from each group and sacrificed by cervical dislocation. The heart, gizzard, liver, kidney, breast, leg, and tibias (left and right) of ducklings were sampled and weighed. The relative weights of organs and carcass were calculated as follows:

Relative organ or carcass weight = (Weight of organ or carcass/Live weight of duckling) × 100(4)

Blood samples were collected into heparinized tubes by cardiac puncture. Plasma was obtained after the removal of red blood cells by centrifugation at 1500× *g* for 15 min. All plasma samples were stored at −20 °C until analysis. The concentrations of blood urea nitrogen (BUN), uric acid (UA), creatinine (CREA), creatine phosphokinase (CPK), glutamate oxaloacetate transaminase (GOT), glutamate pyruvic transaminase (GPT), alkaline phosphatase (ALK), cholinesterase (CHE), total protein (TP), albumin (ALB), and globulin (GLO) in plasma were determined using an automated clinical chemistry analyzer (Automatic Analyzer 7150, Hitachi, Tokyo, Japan), according to the manufacturer’s instructions. The levels of calcium (Ca) and magnesium (Mg) in plasma were determined with flame atomic absorption spectroscopy (Atomic Absorption Spectrophotometer Z-5000, Hitachi, Tokyo, Japan).

### 2.7. Statistical Analysis

A randomized complete block design (RCBD) with a 3 × 2 × 2 factorial arrangement was designed. Treatment groups consisted of three T-2 concentrations (0, 0.5, and 5 mg/kg), two GTP levels (0% and 0.5%), and two duckling breeds (BTD and KD). Data were statistically analyzed using general linear models (GLMs) (PC-SAS^®^ ver. 9.2, 1995) following factorial treatments in a spilt-plot design, in which the two breeds of ducklings nested in each of the 24 pens were regarded as blocks.

The mathematical model is as follows:

*Y_ijkl_* = *μ* + *R_i_* + *T_j_* + *G_k_* + *B_l_* + (*TG*)_*jk*_ + (*TB*)_*jl*_ + (*GB*)_*kl*_ + (*TGB*)_*jkl*_ + *ε_ijkl_* (5)

where *Y_ijkl_* = the observed response of duckling breed in a pen; *μ* = the overall mean; *R_i_* = the effect of the *i*th block (pen); *T_j_* = the fixed effect of T-2 concentrations; *G_k_* = the fixed effect of GTP level; *B_l_* = the fixed effect of duckling breed; (*TG*)*_jk_* = the interaction effect of T-2 concentrations × GTP levels; (*TB*)*_jl_* = the interaction effect of T-2 concentrations × duckling breed; (*GB*)*_kl_* = the interaction effect of GTP levels × duckling breeds; (*TGB*)*_jkl_* = the interaction effect of T-2 concentrations × GTP levels × duckling breeds; and *ε_ijkl_* = the residual error when duckling breed nested in a pen are regarded as an experiment unit, *ε_ijkl_*∩*N* (0, *δ*^2^_*ε*_). Means of the T-2 concentrations, the GTP levels, and the two duckling breeds were compared by using Tukey’s testing, and the significance level was set at *p* < 0.05.

## 3. Results

### 3.1. Antioxidative Capacity of the Green Tea Powder

**Figure 1:**
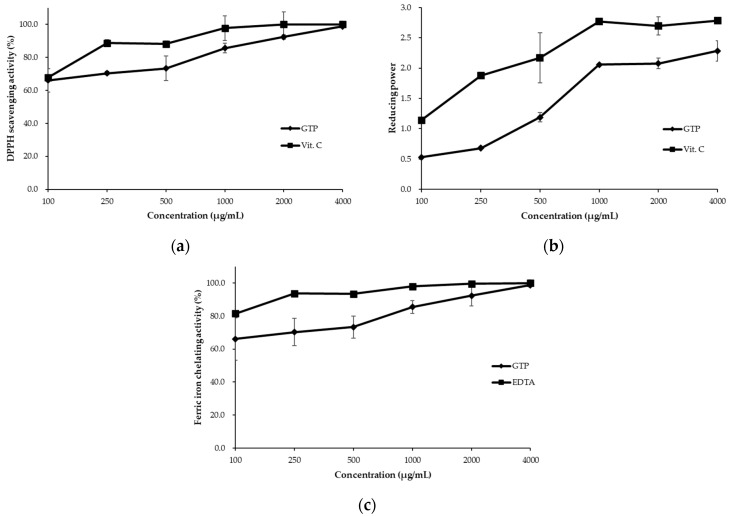
The free radical scavenging capacities of green tea powder (GTP), ascorbic acid (vitamin C, Vit. C), and ethylenediaminetetraacetic acid (EDTA). (**a**) 1,1-diphenyl-2-picrylhydrazyl (DPPH) radical scavenging activity, (**b**) reducing power, and (**c**) ferric ion chelating activity of control (EDTA or Vit. C, ■) and GTP (◆). Data are expressed as the mean ± SD (*n* = 3).

In this study, GTP was used as an antioxidant, and its antioxidative capacity was first tested as shown in Figure 1. The results showed that the DPPH scavenging activity, reducing power, and ferric ion chelating activity of GTP (100–2000 μg/mL) appeared to be lower than those of vitamin C and EDTA. Moreover, the activities of DPPH scavenging and ferric chelating were equivalent to those of the control (vitamin C and EDTA) at a high concentration (4000 μg/mL).

### 3.2. Growth Performance

Breed had a significant effect on the feed intake, BWG, and FCR throughout the experimental period (*p* < 0.0001, Table 3). Daily feed intake and BWG in KDs were higher than in BTDs, but FCR in KDs was lower than that in BTDs. When T-2 concentration was 5 mg/kg, lower feed intake and BWG were observed in duckling when compared to those fed 0 mg/kg of T-2 in the second and third week. The diet contained 0.5% GTP increased BWG and improved FCR in ducklings in the third week. There was an interaction between T-2 concentration and duck breed (*p* < 0.05), wherein the BTDs fed diets contained 5 mg/kg of T-2 had lower feed intake (first week) when compared to the BTDs fed 0 mg/kg of T-2 (Supplementary Materials Table S1). Both ducklings had lower BWG in 5 mg/kg of T-2 treatment in the third week. Only BTDs fed the diet containing 5 mg/kg of T-2 with 0.5% GTP improved the BWG compared to those fed the diet 5 mg/kg without GTP (Table S2). The poor BWG of Kaiya was not improved by GTP in the third week. Additionally, there was an interaction between GTP level and duck on feed intake (second week) and BWG (first week). This interaction induced the significant difference of feed intake between BTDs and KDs in the second week. The diet supplemented 0.5% GTP that increased the feed intake of BTDs, in contrast, it decreased the feed intake of KDs (Table S3). In addition to feed intake, 0.5% GTP-induced KDs had lower BWG in the first week.

### 3.3. Plasma Biochemical Parameters

Unlike the growth performances, breeds had significant effects on BUN, CREA, UA, GOT, GPT, ALB, GLO, and TP (Table 4) in plasma biochemical values. The concentrations of CREA, UA, GOT, GPT, ALB, GLO, and TP in the plasma of KDs were lower than those in BTDs, but BUN in KDs was higher than that in BTDs. The concentrations of Ca, Mg, BUN, CPK, and ALK were affected by dietary T-2 concentrations (*p* < 0.05, Table 4). The concentrations of Ca, CPK, and ALK in the groups treated with 0.5 and 5 mg/kg of T-2 were lower than those without T-2 treatment. Magnesium concentration decreased in the group administered 5 mg/kg of T-2. In contrast to Mg concentration, BUN activity was increased in the blood plasma of the duck fed the diet containing 5 mg/kg of T-2. When the diet was supplemented with 0.5% GTP, lower concentrations of BUN, UA, CHE, GOT, and GPT were observed in the plasma of the ducklings compared to those fed without GTP; however, supplementation with 0.5% GTP increased the plasma concentrations of Ca, GLO, and TP. The BUN concentration of BTD in the group administered 5 mg/kg of T-2 without GTP supplementation was higher than the group not administered T-2 and GTP (Table S4). In addition, KDs fed the diet containing 5 mg/kg of T-2 and 0.5% GTP improved their BUN level compared to those fed with the same diet without GTP. Moreover, CPK activity was also affected by the interactions between T-2 and GTP (*p* < 0.0001, Table S5). Only the ducklings fed the diet without GTP had decreased CPK activity with the increasing T-2 concentrations. Glutamate oxaloacetate transaminase activity was not only associated with the effects of GTP and breed, but it was also under the influence of the two-way interactions between T-2 levels and breed, as well as the three-way interactions of T-2, GTP, and breed (Table 4). Furthermore, GOT activity of BTDs in the group that was administered 5 mg/kg of T-2 without GTP supplementation was higher than the group that had no T-2 treatment (Table S6). Moreover, BTDs fed the diet containing 5 mg/kg of T-2 but supplemented with 0.5% GTP had a reduced GOT level compared to those fed with the same level of T-2 but without GTP supplementation. Additionally, there was an interaction between GTP level and duck breed on ALK activity, which had a significant difference between BTDs and KDs. The diet supplemented with 0.5% GTP decreased ALK activity of BTDs, but increased ALK activity of KDs (Table S7).

### 3.4. Relative Weights of Organs and Carcass

Breed had a significant effect on the relative weights of all organs and carcass examined (*p* < 0.0001, Table 5). In general, organs of BTDs were heavier than those of KDs, but the breasts of KDs were heavier than those of BTDs. The relative weights of gizzards and legs were significantly affected by the effects of T-2 (*p* < 0.05) levels and an interaction between T-2 and breed (*p* < 0.001) was detected. The relative gizzard weight was increased in BTDs in both T-2-treated groups (Table S8). However, the relative leg weight was decreased only in BTDs treated with the highest (5 mg/kg) T-2, compared to the 0 mg/kg of T-2 group. Unlike the BTDs, the relative weights of gizzard and leg were not affected by T-2 concentrations in KDs. It is interesting that T-2 increased relative kidney weight but reduced relative breast weight in the 5 mg/kg of T-2-treated ducklings (Table 5), while those fed with the diet supplemented with 0.5% GTP lowered relative weights of heart, liver, and kidneys. In addition, relative left tibia weight was higher in the ducklings with 0.5% GTP supplementation. There was an interaction between GTP and duck breed (*p* < 0.05); the BTDs fed diets supplemented with 0.5% GTP had higher relative tibia weight (both left and right) when compared to that without GTP (Table S9). In contrast, the relative tibia weights were not affected by GTP supplementation in KDs.

## 4. Discussion

### 4.1. Antioxidative Capacity of Green Tea Powder

Green tea is a traditional Asian beverage and is known for its antioxidative capacity. The current study investigated the antioxidative capacity of GTP by determining the DPPH scavenging capacity, ferric ion chelating activity, and reducing power. A previous study [47] indicated that a positive correlation exists for other antioxidant capacity methods, such as DPPH-scavenging capacity, ferric chelating activity, and reducing power with green tea polyphenols. The GTP of the current study possessed more DPPH scavenging and reducing power than the GTP of other studies. By contrast, ferric ion chelating was lower [23,48]. Several studies have indicated that diets containing 0.5% GTP improved the growth performance, blood biochemical parameters, and the intestinal traits in broilers [49,50]. In addition, our previous tests showed that GTP at 4000 μg/mL had nearly 100% of DPPH radical scavenging activity and ferric ion chelating activity. The concentration of 4000 μg/mL was equivalent to 0.4% GTP supplementation in the duckling feed. Similar to the allowance of T-2 level used, 0.5% but not 0.4% GTP was decided to compensate potential experimental errors in the present study.

### 4.2. Effects of T-2 Toxin and Green Tea Powder on the Growth Performance of Ducklings

In the present study, KDs (meat-type) demonstrated higher feed intake and BWG, and better FCR than BTDs (laying-type). As expected, breed type had a significant impact on the growth performance throughout the experimental period due to the genetics. It has been suggested that meat-type poultry have higher oxidative phosphorylation efficiency than laying-type poultry in their skeletal muscle mitochondria [51]. The basal metabolic rate of meat-type poultry is lower than that of laying-type poultry starting from hatching day until reaching 500 g of body weight [15]. These factors might help explain their better FCR and growth rates. It is known that T-2 could interfere the energy metabolism in poultry [16]. When the ducklings fed the diet containing 5 mg/kg of T-2, lower feed intakes and BWG were observed during the second and the third weeks. Furthermore, another study has suggested that T-2 could suppress the protein synthesis, BWG, and feed intake of chicken and piglets [16]. Interactions between T-2 and breed that decreased the feed intake only in BTDs fed the diet containing 5 mg/kg of T-2 in the first week. In other words, BTDs were more susceptible to T-2 toxicity than KDs in the growth performance. To our best knowledge, no study has been conducted and reported such differential sensitivity between meat- and egg-type ducklings in response to T-2 toxicity. It appears that such diverse physiologic responses are mainly due to the intensive selection and breeding of KDs, although more studies are required. Different from the feed intake and BWG, the FCR of both BTDs and KDs remained unaffected by T-2 toxicity, which is similar to the T-2 effects on White Roman geese [52]. Besides the genetic difference, the reasons behind the indiscernible adverse effects of T-2 toxicity on the growth performance of these ducklings remain unexplained. Nevertheless, supplementation of GTP in duck diets had a significant interaction with breed. The differential breed effects further intensify the GTP inhibition on feed intake and BWG in KDs. A similar phenomenon was also found in broilers, to which 1% GTP was fed, which resulted in reduced feed intakes [53].

It has also been demonstrated that green tea polyphenols could alter the phosphorylation efficiency of mitochondria in rodents [54]. Additionally, our study demonstrated that GTP supplementation also inhibited the toxicity of T-2 (5 mg/kg) in terms of BWG only in BTDs. Therefore, GTP could serve as a detoxicant to protect BTDs against the T-2 toxicity, perhaps in poor growth conditions.

### 4.3. Hematological Alterations after T-2 Toxin Challenge Followed by GTP Supplementation

Hepatic and renal tissues are rich in metabolism-associated enzymes. Damages to these organs often lead to the release of enzymes into the bloodstream, resulting in the elevation of enzymatic activities in peripheral circulation [55,56]. Our analyses showed that T-2 altered plasma biochemical parameters, including hypocalcemia, hypomagnesemia, and low concentrations of CPK and ALK in the blood plasma of both KDs and BTDs. These effects were supported by previous studies, in which blood Ca and Mg concentrations of the intoxicated chicken (treated by 4 mg/kg of T-2) were significantly decreased [57,58]. Other studies have also shown that T-2 induced hypocalcemia by decreasing 1,25(OH)_2_D_3_ receptors in small intestine mucosa [59,60]. When animals show symptoms of hypomagnesemia, parathyroid hormone will be insufficiently produced and hypocalcemia could be further aggravated [61], because Mg is required for the secretion of parathyroid hormone [62].

Alkaline phosphatase plays an important role in the calcification of cartilage and bone via the increased concentration of inorganic phosphates by hydrolyzing phosphate-esters for bone mineralization [63]. Previous studies have demonstrated that diets containing T-2 reduce ALK concentrations in poultry [64,65]. Administration of T-2 can cause hypocalcemia, likely due to a decrease in Ca absorption and inactivation of ALK in small intestine mucosa; in turn, a pathological syndrome, such as spongy bones or osteoporosis, could be observed [60].

Creatine phosphokinase plays an essential role in the energy metabolism of all tissues, particularly in skeletal and cardiac muscles [66]. In contrast to the present study, Edrington et al. [67] indicated that growing broilers fed with T-2 diet (6 mg/kg) for 3 weeks showed no difference in CPK concentrations. It is likely that such different responses in CPK concentrations between ducklings and growing broilers are species-specific (ducks vs. chicken).

Blood urea nitrogen and GOT are two markers that indicate renal injury and liver damage, respectively [68]. In the present study, the treatment of 5 mg/kg of T-2 increased BUN and GOT of the blood plasma in KDs and BTDs, respectively, suggesting the malfunction in both kidneys and livers of the ducklings. It has been reported that broilers that received 3 mg/kg of T-2 for 5 weeks displayed higher concentrations of peripheral GOT and BUN [69]. Another study on broilers treated with only 927 μg/kg of T-2 for 3 weeks caused their bile tract lesions [70]. A similar response was also observed in Japanese quails fed with 4 mg/kg of T-2 for 5 weeks, and an elevation of GOT concentration was detected, with no changes in BUN concentrations [64]. When the diet was supplemented with 0.5% GTP after T-2 treatment (5 mg/kg), an effective improvement of the abnormal BUN and GOT concentrations in the KDs and BTDs, respectively, was observed. Previous studies have indicated that GTP could effectively reduce BUN and GOT concentrations [28,71,72]. Other biochemical parameters, such as the concentrations of GLO and TP, were also increased in ducklings fed a diet supplemented with 0.5% GTP. These results were consistent with previous studies in rats and rainbow trout [73,74]. In general, TP and GLO are involved in several physiological processes, including the transport of ions, hormones, and lipids [75]. The increased concentrations of TP and GLO indicated that GTP might also have played a role in regulating certain physiological mechanisms in ducklings.

### 4.4. Effects of T-2 Toxin and Green Tea Powders on the Relative Weights of Organs and Carcass

In terms of relative organ weight, T-2 induced gizzard enlargement in BTDs, which is supported by previous study in chicken [57]. Furthermore, a decrease in relative breast and leg weights of BTDs was observed when fed with relatively high level of T-2. It might be caused by the altered metabolisms of essential amino acids and the inhibition of protein synthesis by T-2 toxicosis [76,77]. We found that relative kidney weights increased in ducklings fed the diets that contained 0.5 and 5 mg/kg of T-2. However, other studies in broilers [57,67] demonstrated that their relative kidney weights did not differ from the control group, when broilers were fed with 4–6 mg/kg of T-2 for 3 weeks. The differential responses in relative kidney weight between ducklings and broilers remain undetermined. Additionally, it is evident that GTP increased the relative tibia weights (both right and left) in BTDs, which is supported by previous studies [78,79]. Although more in-depth studies are required, the present study found that GTP enhanced the strength of the tibia and was likely also beneficial to growth and bone development.

### 4.5. Detoxification and Antioxidative Activities of Green Tea Powder

It has been reported that T-2 can cause oxidative stress by producing ROS [39,80]. Studies have shown that GTP or green tea extract possesses a strong antioxidant activity, reducing power and scavenging capability on ROS and free radicals [29,81]. The antioxidative effects of GTP are mainly due to its polyphenolic compounds, particularly for the large amount of epigallocatechin (EGC) and epigallocatechin gallate (EGCG) [47]. Epigallocatechin and EGCG, in addition to their antioxidative activity, have also been indicated to possess detoxicant properties against trichothecenes in vitro [82,83]. Other green tea polyphenolic compounds, such as C, CG, EC, ECG, GC, and GCG, also had both antioxidative and DNA protection properties [37]. Several studies have indicated that GTP has the capacity to ameliorate hepatotoxicity and genotoxicity caused by mycotoxins including AFB_1_, FB_1_, or citrinin [29,36,37]. In this study, the improved BWG and GOT or BUN level also confirmed that dietary supplementation of GTP could partially detoxify T-2 toxicity in ducklings.

## 5. Conclusions

In the present study, we have demonstrated that BTDs and KDs had differential responses to T-2 toxicity and GTP detoxification. More sensitive responses to T-2 and GTP supplementation in BTDs were demonstrated in their growth performances, blood plasma parameters, and some organ weights, as well as carcass weights. Moreover, GTP specifically improved the T-2-caused toxicity, including the reversal of hepatic or nephrotic indexes, such as GOT and BUN, in the T-2-treated ducklings. For practical feeding and management of waterfowls, the use of GTP as their dietary supplement can be an option for detoxifying animal consumption of trichothecene contaminated feeds or ingredients.

## Figures and Tables

**Table 1:** Chemical compositions (on dry matter basis) and catechin contents of green tea powder.

Ingredients	Contents
Proximal analysis, g/kg	
Dry matter	989.1
Crude fiber	200.5
Acid detergent fiber	198.3
Neutral detergent fiber	282.6
Green tea polyphenol compositions, mg/100 mL	
Catechin	0.78
Catechin gallate	2.31
Epicatechin	7.16
Epicatechin gallate	2.04
Epigallo catechin	41.93
Epilgallocatechin gallate	20.54
Gallocatechin	3.22
Gallocatechin gallate	0.20

**Table 2:** Ingredients and nutrient compositions of the basal diet (as fed basis).

Ingredient	g/kg
Corn	590
Soybean meal	200
Soybean oil	14.3
Fish meal	50.0
Wheat	60.0
Wheat middling	50.0
Sodium chloride	5.00
Calcium carbonate	9.50
Dicalcium phosphate	14.0
dL-methionine	1.60
L-lysine	8.00
Choline	1.50
Vitamin premix ^a^	2.00
Mineral premix ^b^	1.30
Total	1000
Calculated nutrient composition	
Metabolizable energy, kcal/kg	2913
Crude protein, g/kg	192
Crude fiber, g/kg	36.3
Calcium, g/kg	9.80
Available phosphorus, g/kg	4.60

^a^ Supplied per kg of diet: vitamin A 10,000 IU; vitamin D_3_ 1000 IU; vitamin E 30 mg; vitamin K_3_ 3 mg; vitamin B_2_ 5 mg; vitamin B_6_ 3 mg; vitamin B_12_ 25 mg; folic acid 3 mg; pantothenate 10 mg; niacin 50 mg; biotin 60 mg. ^b^ Supplied per kg of diet: iron 80 mg; copper 10 mg; manganese 55 mg; zinc 60 mg; cobalt 0.4 mg; selenium 0.15 mg.

**Table 3:** Effects of diets containing T-2 toxin and green tea powder on growth performance of Brown Tsaiya ducklings and Kaiya ducklings.

Treatment	Feed Intake, g/bird/day			BWG, g/bird/day			FCR, g/g		
	1st Week	2nd Week	3rd Week	1st Week	2nd Week	3rd Week	1st Week	2nd Week	3rd Week
T-2 toxin (T), mg/kg									
0	25.9	60.3 ^a^	79.5 ^a^	12.7	29.2 ^a^	38.6 ^a^	2.27	2.35	2.21
0.5	26.4	59.2 ^ab^	77.8 ^ab^	12.7	27.2 ^b^	36.0 ^a^	2.44	2.62	2.34
5	25.4	57.3 ^b^	75.2 ^b^	11.7	26.8 ^b^	33.2 ^b^	2.54	2.51	2.45
Green tea powder (G), %									
0	25.8	59.0	77.4	12.6	27.9	33.7 ^b^	2.44	2.46	2.50 ^a^
0.5	26.0	58.6	77.7	12.2	27.5	38.2 ^a^	2.39	2.52	2.17 ^b^
Breed (B)									
BTD ^1^	24.3 ^b^	57.1 ^b^	74.5 ^b^	7.91 ^b^	17.0 ^b^	26.6 ^b^	3.19 ^a^	3.40 ^a^	2.88 ^a^
KD	27.4 ^a^	60.5 ^a^	80.5 ^a^	16.9 ^a^	38.4 ^a^	45.4 ^a^	1.64 ^b^	1.58 ^b^	1.80 ^b^
SEM	0.851	1.293	1.926	0.732	1.156	2.202	0.212	0.162	0.206
Source of variation	*p*-Values								
T	0.2698	0.0141	0.0066	0.0787	0.0101	0.0021	0.2179	0.0774	0.1525
G	0.7052	0.6343	0.7600	0.3423	0.5655	0.0004	0.7104	0.5521	0.0024
B	<0.0001	<0.0001	<0.0001	<0.0001	<0.0001	<0.0001	<0.0001	<0.0001	<0.0001
T × G	0.7839	0.1898	0.2785	0.6109	0.8407	0.7586	0.8118	0.9784	0.5457
T × B	0.0196	0.1997	0.9445	0.2354	0.4165	0.3424	0.5272	0.3158	0.8393
G × B	0.7814	0.0016	0.1823	0.0124	0.6165	0.8757	0.0980	0.2232	0.1688
T × G × B	0.6305	0.3593	0.9224	0.1981	0.6651	0.4780	0.3967	0.7332	0.7325

^a,b^ Means without the same superscripts in the same column differ (*p* < 0.05). ^1^ BTD: Brown Tsaiya duckling; BWG: body weight gain; FCR: feed conversion ratio; KD: Kaiya duckling.

**Table 4:** Effects of diets containing T-2 toxin and green tea powder on plasma biochemical values of Brown Tsaiya ducklings and Kaiya ducklings.

Treatment	Mineral, mg/dL		Renal Function, mg/dL			Liver Function, U/L					Protein, g/dL		
	Ca	Mg	BUN	CREA	UA	CPK	CHE	GOT	GPT	ALK	ALB ^1^	GLO	TP
T-2 toxin (T), mg/kg													
0	10.89 ^a^	9.39 ^a^	3.34 ^b^	0.171	3.57	2787 ^a^	2911	56.0	44.2	458 ^a^	1.50	1.60	3.08
0.5	8.26 ^b^	8.21 ^a^	3.41 ^ab^	0.159	2.86	2315 ^b^	2878	65.6	45.9	373 ^b^	1.49	1.55	3.06
5	6.73 ^c^	6.76 ^b^	3.75 ^a^	0.166	3.62	2188 ^b^	2929	68.9	41.8	356 ^b^	1.41	1.54	2.94
Green tea powder (G), %													
0	8.04 ^b^	8.25	3.60 ^a^	0.165	3.78 ^a^	2439	3042 ^a^	70.1 ^a^	44.7	410	1.42	1.51 ^b^	2.92 ^b^
0.5	9.21 ^a^	7.99	3.40 ^b^	0.166	2.93 ^b^	2421	2769 ^b^	56.9 ^b^	43.3	382	1.51	1.62 ^a^	3.13 ^a^
Breed (B)													
BTD	8.55	8.06	3.15 ^b^	0.175 ^a^	3.81 ^a^	2421	2913	71.7 ^a^	48.1 ^a^	399	1.65 ^a^	1.74 ^a^	3.39 ^a^
KD	8.70	8.18	3.85 ^a^	0.155 ^b^	2.89 ^b^	2439	2899	55.3 ^b^	39.8 ^b^	393	1.28 ^b^	1.38 ^b^	2.66 ^b^
SEM	0.655	0.744	0.214	0.011	0.705	154	74.7	9.99	3.44	31.1	0.081	0.073	0.149
Source of variation	*p*-Values												
T	<0.0001	<0.0001	0.0188	0.3407	0.2375	<0.0001	0.6113	0.1718	0.2552	<0.0001	0.2061	0.4324	0.3877
G	0.0028	0.5491	0.0456	0.8218	0.0396	0.8436	<0.0001	0.0248	0.5036	0.1293	0.0601	0.0108	0.0211
B	0.6869	0.7708	<0.0001	0.0025	0.0275	0.8451	0.7337	0.0058	<0.0001	0.7522	<0.0001	<0.0001	<0.0001
T × G	0.5745	0.8717	0.1829	0.3174	0.0039	<0.0001	0.8452	0.3352	0.5517	0.3592	0.9189	0.5207	0.9378
T × B	0.2801	0.6945	0.8197	0.8335	0.5316	0.9816	0.0587	0.0123	0.4972	0.9911	0.8835	0.3255	0.6349
G × B	0.9308	0.8524	0.1810	0.3519	0.9959	0.4574	0.7270	0.1390	0.8342	0.0080	0.8945	0.8829	0.9807
T × G × B	0.1637	0.9475	0.3536	0.8707	0.6821	0.7940	0.4463	0.0186	0.8984	0.7763	0.8835	0.6429	0.8202

^a–c^ Means without the same superscripts in the same column differ (*p* < 0.05). ^1^ ALB: albumin; ALK: alkaline phosphatase; BTD: Brown Tsaiya duckling; BUN: blood urea nitrogen; Ca: calcium; CREA: creatinine; CPK: creatine phosphokinase; CHE: cholinesterase; GOT: glutamate oxaloacetate transaminase; GPT: glutamic pyruvic transaminase; GLO: globulin; KD: Kaiya duckling; Mg: magnesium; TP: total protein; UA: uric acid.

**Table 5:** Effects of diets containing T-2 toxin and green tea powder on the relative weights of organs and carcass of Brown Tsaiya ducklings and Kaiya ducklings.

Treatment	g/100 g BW							
	Organs				Carcass			
	Heart	Gizzard	Liver	Kidney	Breast	Leg	Left Tibia	Right Tibia
T-2 toxin (T), mg/kg								
0	0.921	5.75 ^b^	3.56	1.13 ^b^	0.917 ^a^	7.16 ^a^	1.56	1.49
0.5	0.961	8.18 ^a^	3.73	1.27 ^a^	0.855 ^ab^	6.76 ^ab^	1.51	1.54
5	0.983	8.54 ^a^	3.86	1.29 ^a^	0.763 ^b^	6.34 ^b^	1.44	1.53
Green tea powder (G), %								
0	0.993 ^a^	7.47	3.92 ^a^	1.29 ^a^	0.823	6.53	1.42 ^a^	1.48
0.5	0.917 ^b^	7.51	3.51 ^b^	1.18 ^b^	0.866	6.98	1.58 ^b^	1.55
Breed (B)								
BTD ^1^	1.141 ^a^	9.26 ^a^	4.21 ^a^	1.58 ^a^	0.709 ^b^	7.85 ^a^	1.85 ^a^	1.89 ^a^
KD	0.769 ^b^	5.72 ^b^	3.22 ^b^	0.89 ^b^	0.981 ^a^	5.66 ^b^	1.16 ^b^	1.15 ^b^
SEM	0.051	0.405	0.215	0.084	0.081	0.431	0.102	0.114
Source of variation	*p*-Values							
T	0.2142	<0.0001	0.1458	0.0127	0.0286	0.0294	0.2615	0.1062
G	0.0098	0.8687	0.0012	0.0254	0.3609	0.0773	0.0088	0.3016
B	<0.0001	<0.0001	<0.0001	<0.0001	<0.0001	<0.0001	<0.0001	<0.0001
T × G	0.3407	0.5519	0.7071	0.2755	0.8118	0.5560	0.6880	0.7386
T × B	0.2282	<0.0001	0.0663	0.8104	0.3007	0.0032	0.0597	0.5375
G × B	0.0720	0.4704	0.0842	0.1710	0.0810	0.1093	0.0056	0.0424
T × G × B	0.6305	0.3593	0.9224	0.1981	0.6651	0.9003	0.3967	0.7332

^a,b^ Means without the same superscripts in the same column differ (*p* < 0.05). ^1^ BTD: Brown Tsaiya duckling; BW: body weight; KD: Kaiya duckling.

## Data Availability

Data available in a publicly accessible repository.

## Supplementary Materials

The following are available online at https://www.mdpi.com/article/10.3390/ani11092541/s1, Table S1: Feed intake of Brown Tsaiya ducklings and Kaiya ducklings fed diets containing different concentrations of T-2 toxin (T-2) during the first week. Table S2: Body weight gain of Brown Tsaiya ducklings and Kaiya ducklings fed diets containing different concentrations of T-2 toxin (T-2) with or without green tea powder (GTP) supplementation during the third week. Table S3: Feed intake (second week) and body weight gain (first week) of Brown Tsaiya ducklings and Kaiya ducklings fed diets supplemented with or without green tea powder (GTP). Table S4: Blood urea nitrogen concentrations of Brown Tsaiya ducklings and Kaiya ducklings fed diets containing different concentrations of T-2 toxin (T-2) with or without green tea powder (GTP) supplementation. Table S5: Creatine phosphokinase activity of ducklings fed diets containing different T-2 toxin (T-2) concentrations with or without green tea powder. Table S6: Glutamate oxaloacetate transaminase activity of Brown Tsaiya ducklings and Kaiya ducklings fed diets containing different concentrations of T-2 toxin (T-2) with or without green tea powder (GTP). Table S7: Alkaline phosphatase activity of Brown Tsaiya ducklings and Kaiya ducklings fed diets with or without green tea powder (GTP). Table S8: The relative weights of gizzard and leg of Brown Tsaiya ducklings and Kaiya ducklings fed diets containing different T-2 toxin (T-2) concentrations. Table S9: The relative weights of tibias (left and right) of Brown Tsaiya ducklings and Kaiya ducklings fed diets with or without green tea powder (GTP).
