# Supplementary material for: Differential Effects of Green Tea Powders on the Protection of Brown Tsaiya and Kaiya Ducklings against Trichothecene T-2 Toxin Toxicity
Source: Animals (Basel). 2021 Aug 30;11(9):2541. doi: 10.3390/ani11092541 (PMC8466186; doi:10.3390/ani11092541)
Supplement: Supplementary file 1 [file animals-11-02541-s001.zip › animals-1268023-supplementary.pdf]

animals

MDPI

Supplementary Table S1. Feed intake of Brown Tsaiya ducklings and Kaiya ducklings fed diets containing different T-2 toxin (T-2) concentrations during the first week

Breed	Feed intake, g/bird/day		
	0 mg/kg T-2	0.5 mg/kg T-2	5 mg/kg T-2
Brown Tsaiya ducklings	25.1 ^{a}	25.1 ^{aB}	22.9 ^{bB}
Kaiya ducklings	26.7	27.6 ^{A}	27.9 ^{A}

$^{a,b/A,B}$Means followed by small capital letters, in the same row, and distinct letters, in the same column, are different ($p \leq 0.05$) by Tukey's test.

Supplementary Table S2. Body weight gain of Brown Tsaiya ducklings and Kaiya ducklings fed diets containing different concentrations of T-2 toxin (T-2) concentrations with or without green tea powder (GTP) during the third week

Breed	Body weight gain, g/bird/day					
	0 mg/kg T-2		0.5 mg/kg T-2		5 mg/kg T-2	
	0% GTP	0.5% GTP	0% GTP	0.5% GTP	0% GTP	0.5% GTP
Brown Tsaiya ducklings	26.0 ^{aB}	30.5 ^{aB}	25.0 ^{aB}	28.0 ^{aB}	22.2 ^{bB}	27.7 ^{aB}
Kaiya ducklings	47.9 ^{abA}	50.1 ^{aA}	42.0 ^{cA}	49.2 ^{abA}	39.2 ^{cA}	43.9 ^{bcA}

$^{a-c/A,B}$Means followed by small capital letters, in the same row, and distinct letters, in the same column, are different ($p \leq 0.05$) by Tukey's test.

Supplementary Table S3. Feed intake (second week) and body weight gain (first week) of Brown Tsaiya ducklings and Kaiya ducklings fed diets supplemented with or without green tea powder (GTP)

Breed	Feed intake, g/bird/day, 2nd week		Body weight gain, g/bird/day, 1st week	
	0% GTP	0.5 % GTP	0% GTP	0.5 % GTP
Brown Tsaiya ducklings	56.0 ^{bB}	58.2 ^{a}	7.53 ^{B}	8.24 ^{B}
Kaiya ducklings	62.0 ^{aA}	59.1 ^{b}	17.6 ^{aA}	16.1 ^{bA}

$^{a,b/A,B}$Means followed by small capital letters, in the same row, and distinct letters, in the same column, are different ($p \leq 0.05$) by Tukey's test.

Supplementary Table S4. Blood urea nitrogen concentrations of Brown Tsaiya ducklings and Kaiya ducklings fed diets containing different concentrations of T-2 toxin (T-2) with or without green tea powder (GTP) supplementation

Breed	Blood urea nitrogen, mg/dL					
	0 mg/kg T-2		0.5 mg/kg T-2		5 mg/kg T-2	
	0% GTP	0.5% GTP	0% GTP	0.5% GTP	0% GTP	0.5% GTP
Brown Tsaiya ducklings	3.00 ^{B}	3.00 ^{B}	3.00 ^{B}	3.00 ^{B}	3.50 ^{B}	3.38
Kaiya ducklings	3.63 ^{bA}	3.75 ^{bA}	4.00 ^{abA}	3.63 ^{bA}	4.50 ^{aA}	3.63 ^{b}

$^{a,b/A,B}$Means followed by small capital letters, in the same row, and distinct letters, in the same column, are different ($p \leq 0.05$) by Tukey's test.

Supplementary Table S5. Creatine phosphokinase activity of ducklings fed diets containing different T-2 toxin (T-2) concentrations with or without green tea powder

Green tea powder, %	Creatine phosphokinase, U/L		
	0 mg/kg T-2	0.5 mg/kg T-2	5 mg/kg T-2
0	3074 aA	2244 b	1998 cB
0.5	2499 B	2386	2378 A

a,b,c/ A,B Means followed by small capital letters, in the same row, and distinct letters, in the same column, are different ($p \leq 0.05$) by Tukey's test.

Supplementary Table S6. Glutamate oxaloacetate transaminase activity of Brown Tsaiya ducklings and Kaiya ducklings fed diets containing different concentrations of T-2 toxin (T-2) with or without green tea powder (GTP)

Breed	Glutamate oxaloacetate transaminase, U/L					
	0 mg/kg T-2		0.5 mg/kg T-2		5 mg/kg T-2	
	0% GTP	0.5% GTP	0% GTP	0.5% GTP	0% GTP	0.5% GTP
Brown Tsaiya ducklings	56.0 b	58.8 b	76.0 b	60.3 b	115.8 aA	63.3 b
Kaiya ducklings	66.3	42.9	59.6	66.5	46.9 B	49.6

a,b/ A,B Means followed by small capital letters, in the same row, and distinct letters, in the same column, are different ($p \leq 0.05$) by Tukey's test.

Supplementary Table S7. Alkaline phosphatase activity of Brown Tsaiya ducklings and Kaiya ducklings fed diets with or without green tea powder (GTP)

Breed	Alkaline phosphatase activity, U/L	
	0% GTP	0.5 % GTP
Brown Tsaiya ducklings	437 aA	361 bB
Kaiya ducklings	383 bB	404 aA

a,b/ A,B Means followed by small capital letters, in the same row, and distinct letters, in the same column, are different ($p \leq 0.05$) by Tukey's test.

Supplementary Table S8. The relative weights of gizzard and leg of Brown Tsaiya ducklings and Kaiya ducklings fed diets containing different T-2 toxin (T-2) concentrations

	Gizzard, g/100 g body weight			Leg, g/100 g body weight		
	0 mg/kg T-2	0.5 mg/kg T-2	5 mg/kg T-2	0 mg/kg T-2	0.5 mg/kg T-2	5 mg/kg T-2
Brown Tsaiya ducklings	6.00 b	10.7 aA	11.1 aA	8.71 aA	8.00 aA	6.86 bA
Kaiya ducklings	5.49	5.69 B	5.98 B	5.61 B	5.53 B	5.83 B

a,b/ A,B Means followed by small capital letters, in the same row, and distinct letters, in the same column, are different ($p \leq 0.05$) by Tukey's test.

Supplementary Table S9. The relative weights of tibias (left and right) of Brown Tsaiya ducklings and Kaiya ducklings fed diets with or without green tea powder (GTP)

Breed	Left tibia, g/100 g body weight		Right tibia, g/100 g body weight	
	0% GTP	0.5 % GTP	0 % GTP	0.5 % GTP
Brown Tsaiya ducklings	1.68 bA	2.01 aA	1.79 bA	1.99 aA
Kaiya ducklings	1.16 B	1.16 B	1.18 B	1.11 B

a,b/ A,B Means followed by small capital letters, in the same row, and distinct letters, in the same column, are different ($p \leq 0.05$) by Tukey's test.

© 2021 by the authors. Submitted for possible open access publication under the terms and conditions of the Creative Commons Attribution (CC BY) license (http://creativecommons.org/licenses/by/4.0/).
